# Family Medicine and Emergency Physicians’ Perceptions of Inappropriate Emergency Room Use in Saudi Arabia: A Cross-Sectional Survey

**DOI:** 10.7759/cureus.105149

**Published:** 2026-03-13

**Authors:** Najlaa M Alsudairy, Deemah A Altashkandi, Alaa O Alahdal, Fatmah S Baqar

**Affiliations:** 1 Department of Family Medicine, Ministry of the National Guard - Health Affairs, Jeddah, SAU; 2 Department of Family Medicine, King Abdullah International Medical Research Center, Jeddah, SAU; 3 College of Medicine, King Saud Bin Abdulaziz University for Health Sciences, Jeddah, SAU

**Keywords:** crowding, emergency medicine, emergency room utilization, family medicine, healthcare costs, nonurgent care, patient safety, primary care, referral systems, saudi arabia

## Abstract

Background: Inappropriate emergency room (ER) utilization for nonurgent conditions is increasingly recognized as a challenge for health systems, contributing to crowding, delays in care, and increased healthcare costs. Physicians working in emergency and primary care settings may have different perspectives on the magnitude, causes, and consequences of this problem. This study assessed physicians’ perceptions of inappropriate ER utilization in Saudi Arabia and compared perspectives between family medicine and emergency medicine physicians.

Methods: We conducted a national cross-sectional, web-based survey of family medicine and emergency medicine physicians practicing in Saudi Arabia in January and February 2026. The questionnaire assessed perceptions of inappropriate ER use, perceived contributing factors, and perceived impacts on care delivery and workforce outcomes. Comparisons between specialties were performed using chi-square tests. Multivariable logistic regression was used to identify factors associated with reporting that at least half of ER visits were inappropriate.

Results: A total of 412 physicians completed the survey, including 214 family medicine physicians (51.9%) and 198 emergency physicians (48.1%). Overall, 352 physicians (85.4%) agreed that inappropriate ER use is a major national problem. A large proportion of physicians reported that many ER visits are nonurgent (n=334, 81.1%) and could be managed in primary care settings (n=318, 77.2%). Emergency physicians were more likely than family physicians to report that ER visits are frequently nonurgent (174 of 198 (87.9%) vs. 160 of 214 (74.8%), P<0.001) and manageable in primary care (176 of 198 (88.9%) vs. 142 of 214 (66.4%), P<0.001). Limited access to primary care was identified as a contributing factor by 304 physicians (73.8%), and after-hours service gaps by 286 (69.4%). A total of 346 physicians (84.0%) agreed that inappropriate ER use delays care for critically ill patients, and 328 (79.6%) agreed that it negatively affects patient safety. In multivariable analysis, emergency physicians were more likely than family physicians to report that at least half of ER visits were inappropriate (adjusted odds ratio (aOR), 2.14; 95% confidence interval (CI), 1.38-3.31; P=0.001). Perceived weaknesses in the referral system (aOR, 2.78; 95% CI, 1.84-4.19; P<0.001) and limited primary care access (aOR, 1.89; 95% CI, 1.23-2.91; P=0.004) were also independently associated with this perception.

Conclusion: In this survey of physicians in Saudi Arabia, inappropriate ER utilization was widely perceived as common and consequential for healthcare delivery. Emergency physicians were more likely than family physicians to characterize ER visits as frequently inappropriate and to report negative impacts on patient safety and care delivery. Strengthening primary care access and referral systems may help reduce nonurgent ER visits and improve the efficiency of emergency services.

## Introduction

Emergency rooms (ERs) play a critical role in providing timely care for acute and life-threatening conditions [1-4]. However, the use of ER services for conditions that could be managed in primary care settings has increasingly been recognized as a challenge for health systems worldwide [1-4]. Such visits, often described as nonurgent or inappropriate ER utilization, contribute to overcrowding, prolonged waiting times, and increased healthcare costs, while potentially delaying care for patients with serious conditions [2,3]. Persistent ER crowding has also been associated with adverse effects on patient safety, quality of care, and clinician workload [3-6].

Multiple factors contribute to the use of ER services for nonurgent conditions [5-9]. Patient-level drivers include limited understanding of appropriate care pathways, anxiety regarding symptoms, and perceptions that ERs offer faster or more comprehensive care [3,6]. System-level factors may also play an important role, including barriers to timely primary care access, limited after-hours services, and weak referral systems between primary care and hospital-based services [3,7]. Financial structures and the absence of cost barriers in some health systems may further encourage ER use for conditions that might otherwise be managed in outpatient settings [5,8]. Understanding the relative contribution of these factors is important for designing effective policy and organizational responses.

Physicians working in both emergency medicine and family medicine are uniquely positioned to observe patterns of ER utilization and their underlying causes [6-13]. Emergency physicians frequently encounter patients presenting with conditions that may not require emergency care, whereas family physicians are often responsible for providing comprehensive and continuous care in community settings [8-11]. Differences in clinical environments and professional responsibilities may therefore influence how these groups perceive the magnitude, causes, and consequences of inappropriate ER use. Despite the importance of these perspectives, comparative data on physicians’ perceptions across specialties remain limited [4,9,13].

In the Kingdom of Saudi Arabia, ongoing health system reforms aim to strengthen primary care and improve the efficiency of service delivery. Although ER services are widely accessible and commonly used, concerns have been raised about high volumes of nonurgent visits and their impact on care delivery. Understanding how physicians perceive the drivers and consequences of inappropriate ER utilization may help inform strategies to improve care coordination, optimize the use of emergency services, and support broader health system reforms.

## Materials and methods

Study design

We conducted a cross-sectional, web-based survey of family medicine and emergency medicine physicians practicing in the Kingdom of Saudi Arabia in January and February 2026. The study was designed to assess physicians’ perceptions of inappropriate ER utilization and its determinants. Participation was voluntary and anonymous, and electronic informed consent was obtained from all respondents before survey initiation. The study was approved by the Standing Committee for Sabbatical Leaves, Publication and Research Ethics, Ministry of Health, Kingdom of Saudi Arabia (registration number: REC-2025-17483).

Eligibility criteria and sampling strategy

Eligible participants were licensed physicians currently practicing in family medicine or emergency medicine in Saudi Arabia, including residents, specialists, and consultants in public or private sectors. Physicians not actively engaged in clinical practice during the study period were excluded.

Participants were recruited through professional networks, institutional mailing lists, and specialty-specific social media groups. The survey link was distributed electronically using a nonprobability convenience sampling approach. To minimize duplicate responses, the survey platform restricted multiple submissions from the same device. Only fully completed questionnaires were included in the final analysis.

Sample size determination

The target sample size was calculated on the basis of detecting a 15-percentage-point difference between family physicians and emergency physicians in the proportion perceiving ER visits as frequently inappropriate, with 80% power and a two-sided alpha level of 0.05. Assuming equal group sizes, a minimum of 356 participants was required; to account for incomplete responses, we aimed to recruit at least 400 participants.

Survey development and validation

The questionnaire items were structured to assess demographic characteristics, perceptions of inappropriate ER use, perceived causes, impact on care delivery and workforce, and potential solutions (see Appendices). Most perception items were measured on a five-point Likert scale (ranging from “strongly disagree” to “strongly agree”). For primary analyses, Likert responses were prespecified to be dichotomized into “agree or strongly agree” versus all other responses. The survey also included multiple-response items regarding perceived causes and one outcome item assessing whether physicians believed that at least 50% of ER visits were inappropriate.

Content validity was assessed by three senior consultants in family medicine and emergency medicine, who reviewed the questionnaire for clarity, relevance, and comprehensiveness. A pilot test was conducted among 15 physicians to evaluate clarity and completion time; minor wording modifications were made accordingly. Internal consistency of perception domains was assessed using Cronbach’s alpha.

Data collection procedures

The survey was administered through a secure online platform. The first page included study information and an electronic consent statement. No personally identifiable information was collected. Data were stored in password-protected files accessible only to the study investigators. Responses were exported into a statistical software package for analysis.

Outcome measures

The primary outcome was the proportion of physicians who perceived inappropriate ER use as a major national problem. A key secondary outcome was the proportion of physicians reporting that at least 50% of ER visits were inappropriate. Additional outcomes included perceived causes of inappropriate utilization and perceived impact on patient safety, delays in care, healthcare costs, and physician burnout.

Statistical analysis

Continuous variables are reported as means ± standard deviations (SDs), and categorical variables as counts and percentages. Comparisons between family medicine physicians and emergency physicians were performed using the chi-square test or Fisher’s exact test for categorical variables and the independent-samples t-test for continuous variables, as appropriate. All tests were two-sided, and a P value of less than 0.05 was considered to indicate statistical significance.

Multivariable logistic regression analysis was performed to identify factors independently associated with reporting that at least 50% of ER visits were inappropriate. Covariates included specialty (emergency vs. family medicine), years of experience (≥10 vs. <10 years), practice sector (government vs. non-government), and agreement with statements regarding limited primary care access and weak referral systems. Adjusted odds ratios (aORs) with 95% confidence intervals (CIs) were calculated. All statistical analyses were conducted using IBM SPSS Statistics for Windows, version 27 (IBM Corp., Armonk, New York, United States).

## Results

Participant characteristics

A total of 412 physicians completed the survey, including 214 family medicine physicians (51.9%) and 198 emergency physicians (48.1%). The mean (±SD) age of participants was 37.6±8.9 years. Overall, 176 participants (42.7%) were women, and 298 (72.3%) were Saudi nationals. Most respondents (n=302, 73.3%) practiced in government-sector institutions. The mean duration of clinical experience was 9.8±6.4 years, and 148 participants (35.9%) were consultants. A higher proportion of women were in family medicine (104 of 214, 48.6%) than in emergency medicine (72 of 198, 36.4%), and a higher proportion of emergency physicians were non-Saudi (68 of 198, 34.3%) compared with family medicine physicians (46 of 214, 21.5%) (Table 1).

**Table 1:** Characteristics of the surveyed physicians (N=412) *Consultant level refers to physicians holding a consultant appointment at the time of the survey. Percentages may not total 100 because of rounding.

Characteristic		Total (N=412)	Family Medicine (n=214)	Emergency Medicine (n=198)
Age (year), n (%)	<30	86 (20.9)	48 (22.4)	38 (19.2)
	30–39	174 (42.2)	92 (43.0)	82 (41.4)
	40–49	98 (23.8)	50 (23.4)	48 (24.2)
	≥50	54 (13.1)	24 (11.2)	30 (15.2)
Female sex, n (%)		176 (42.7)	104 (48.6)	72 (36.4)
Saudi nationality, n (%)		298 (72.3)	168 (78.5)	130 (65.7)
Duration of practice (years), mean ± SD		9.8 ± 6.4	10.4 ± 6.8	9.1 ± 6.0
Consultant level*, n (%)		148 (35.9)	78 (36.4)	70 (35.4)
Government sector, n (%)		302 (73.3)	150 (70.1)	152 (76.8)

Perceptions of inappropriate ER utilization

Overall, 352 physicians (85.4%) agreed or strongly agreed that inappropriate ER use is a major national problem. Emergency physicians were more likely than family physicians to endorse this perception (178 of 198 (89.9%) vs. 174 of 214 (81.3%), P=0.01). A total of 334 respondents (81.1%) agreed that a large proportion of ER visits are nonurgent, with greater agreement among emergency physicians (174 of 198, 87.9%) than among family physicians (160 of 214, 74.8%) (P<0.001). Similarly, 318 physicians (77.2%) reported that many ER cases could be managed in primary care settings; this view was more common among emergency physicians (176 of 198, 88.9%) than among family physicians (142 of 214, 66.4%) (P<0.001). In contrast, family physicians were more likely to attribute inappropriate ER use to limited access to primary care services (170 of 214 (79.4%) vs. 126 of 198 (63.6%), P=0.002). The perception that patients prefer ER care because it is faster and more comprehensive was common overall (n=338, 82.0%), without a significant difference between specialties (Table 2).

**Table 2:** Physicians’ perceptions of inappropriate emergency room utilization (N=412) "Agree" includes responses of “agree” or “strongly agree” on a five-point Likert scale. P values were calculated for comparisons between specialties using the chi-square test. The χ² statistic is shown for each comparison. A P value of <0.05 was considered statistically significant, and statistically significant results are indicated with an asterisk (*). FMP: family medicine physician; EP: emergency physician

Statement	Total Agree, n (%)	FMP (n=214) Agree, n (%)	EP (n=198) Agree, n (%)	χ² value	P Value
Inappropriate ER use is a major national problem	352 (85.4)	174 (81.3)	178 (89.9)	χ² = 6.51	0.01*
Large proportion of ER visits are non-urgent	334 (81.1)	160 (74.8)	174 (87.9)	χ² = 12.47	<0.001*
Many ER cases could be managed in primary care	318 (77.2)	142 (66.4)	176 (88.9)	χ² = 29.84	<0.001*
Limited primary care access contributes to ER overuse	296 (71.8)	170 (79.4)	126 (63.6)	χ² = 12.30	0.002*
Patients perceive ER as faster and more comprehensive	338 (82.0)	180 (84.1)	158 (79.8)	χ² = 1.23	0.27

Perceived causes of inappropriate ER utilization

When asked to identify contributing factors (multiple responses permitted), 304 physicians (73.8%) cited limited access to primary care, and 286 (69.4%) identified after-hours service gaps. Limited access was more frequently reported by family physicians (174 of 214, 81.3%) than by emergency physicians (130 of 198, 65.7%) (P<0.001). Emergency physicians were more likely to identify patient anxiety (134 of 198 (67.7%) vs. 118 of 214 (55.1%), P=0.01) and the absence of financial disincentives for ER visits (130 of 198 (65.7%) vs. 108 of 214 (50.5%), P=0.002) as major contributors. A weak referral system was cited by 264 respondents (64.1%) and was more frequently reported by family physicians (152 of 214, 71.0%) than by emergency physicians (112 of 198, 56.6%) (P=0.003) (Table 3).

**Table 3:** Physicians’ reported causes of inappropriate emergency room utilization (N=412) Values represent the number (percentage) of respondents who identified each factor as contributing to inappropriate emergency room (ER) utilization. Multiple responses were permitted. Comparisons between specialties were performed using the chi-square test, and the χ² statistic is shown for each comparison. A P value of <0.05 was considered statistically significant, and statistically significant values are indicated with an asterisk (*). FMP: family medicine physician; EP: emergency physician

Cause	Total, n (%)	FMP (n=214), n (%)	EP (n=198), n (%)	χ² value	P Value
Limited access to primary care	304 (73.8)	174 (81.3)	130 (65.7)	χ² = 12.58	<0.001*
After-hours service gaps	286 (69.4)	150 (70.1)	136 (68.7)	χ² = 0.09	0.76
Poor health literacy	278 (67.5)	140 (65.4)	138 (69.7)	χ² = 0.81	0.37
Patient anxiety	252 (61.2)	118 (55.1)	134 (67.7)	χ² = 6.62	0.01*
Free ER services	238 (57.8)	108 (50.5)	130 (65.7)	χ² = 9.45	0.002*
Weak referral system	264 (64.1)	152 (71.0)	112 (56.6)	χ² = 8.76	0.003*

Perceived impact on care delivery and workforce

A total of 346 physicians (84.0%) agreed that inappropriate ER use delays care for critically ill patients, and 328 (79.6%) agreed that it negatively affects patient safety. These perceptions were more pronounced among emergency physicians than among family physicians (182 of 198 (91.9%) vs. 164 of 214 (76.6%) for delays in care; 174 of 198 (87.9%) vs. 154 of 214 (72.0%) for patient safety; P<0.001 for both comparisons). Overall, 294 respondents (71.4%) reported that inappropriate ER utilization contributes to physician burnout, with higher agreement among emergency physicians (152 of 198, 76.8%) than among family physicians (142 of 214, 66.4%) (P=0.02). In addition, 336 physicians (81.6%) agreed that inappropriate ER use increases healthcare costs, without a significant difference between specialties (Table 4).

**Table 4:** Physicians’ perceptions of the impact of inappropriate emergency room utilization on care delivery and workforce outcomes (N=412) "Agree" includes responses of “agree” or “strongly agree” on a five-point Likert scale. A P value of <0.05 was considered statistically significant, and statistically significant results are indicated with an asterisk (*). FMP: family medicine physician; EP: emergency physician

Outcome	Total Agree, n (%)	FMP (n=214) Agree, n (%)	EP (n=198) Agree, n (%)	χ² value	P Value
Increases physician burnout	294 (71.4)	142 (66.4)	152 (76.8)	χ² = 5.48	0.02*
Delays care for critical patients	346 (84.0)	164 (76.6)	182 (91.9)	χ² = 17.83	<0.001*
Negatively affects patient safety	328 (79.6)	154 (72.0)	174 (87.9)	χ² = 15.71	<0.001*
Increases healthcare costs	336 (81.6)	170 (79.4)	166 (83.8)	χ² = 1.12	0.29

Factors associated with perceiving ER visits as frequently inappropriate

In multivariable logistic-regression analysis, emergency physicians were more than twice as likely as family physicians to report that at least half of ER visits are inappropriate (aOR, 2.14; 95% CI, 1.38 to 3.31; P=0.001). Perception of a weak referral system was strongly associated with this outcome (aOR, 2.78; 95% CI, 1.84 to 4.19; P<0.001), as was the belief that primary care access is limited (aOR, 1.89; 95% CI, 1.23 to 2.91; P=0.004). Physicians with 10 or more years of experience were modestly more likely to classify ER visits as frequently inappropriate (aOR, 1.42; 95% CI, 1.01 to 2.02; P=0.045). Practice in the government sector was not independently associated with this perception (Table 5).

**Table 5:** Factors associated with physicians’ perception that emergency room visits are frequently inappropriate Shown are the results of a multivariable logistic regression analysis examining factors associated with physicians reporting that at least 50% of emergency room (ER) visits are inappropriate. Perceptions regarding a weak referral system and limited primary care access were derived from agreement with corresponding survey statements. P values are two-sided. The Wald statistic for each predictor is shown. A P value of <0.05 was considered statistically significant, and statistically significant results are indicated with an asterisk (*).

Variable	Adjusted Odds Ratio (95% CI)	Wald statistic	P Value
Emergency physician (vs family physician)	2.14 (1.38–3.31)	10.84	0.001*
≥10 years experience	1.42 (1.01–2.02)	4.02	0.045*
Government sector practice	1.36 (0.88–2.09)	1.96	0.16
Perceived weak referral system	2.78 (1.84–4.19)	21.36	<0.001*
Belief that primary care access is limited	1.89 (1.23–2.91)	8.30	0.004*

## Discussion

In this survey of family medicine and emergency medicine physicians, a large majority of respondents perceived inappropriate ER utilization as a major problem. More than four-fifths of physicians reported that a substantial proportion of ER visits are nonurgent and could be managed in primary care settings. Emergency physicians were consistently more likely than family physicians to characterize ER visits as frequently inappropriate and to report negative consequences for patient safety, delays in care, and physician workload. These findings suggest that inappropriate ER use is widely recognized by frontline physicians and is perceived as having important implications for both health system efficiency and clinical care.

Differences between specialties were notable. Emergency physicians were significantly more likely than family physicians to report that many ER visits are nonurgent and can be managed in primary care. This difference may reflect the clinical context in which emergency physicians encounter a large volume of patients presenting with low-acuity conditions, which may heighten awareness of potentially avoidable visits. In contrast, family physicians were more likely to attribute ER overuse to structural limitations within the primary care system, particularly restricted access to appointments and gaps in after-hours services. These differences in perspective likely reflect the distinct roles and clinical environments of the two specialties and highlight the multifactorial nature of ER utilization patterns [13-16].

The study also identified several perceived drivers of inappropriate ER use. Limited access to primary care services was the most frequently reported contributing factor, particularly among family physicians. After-hours service gaps, poor health literacy, and a weak referral system were also commonly cited. These findings suggest that structural barriers within the health system may play a substantial role in shaping care-seeking behavior. At the same time, emergency physicians more often identified patient anxiety and the absence of financial disincentives for ER visits as contributing factors, indicating that patient preferences and health system incentives may also influence utilization patterns [7,10].

Physicians in this study widely perceived inappropriate ER use as having meaningful consequences for care delivery. Most respondents reported that such utilization delays care for critically ill patients and negatively affects patient safety, perceptions that were especially common among emergency physicians. These concerns are consistent with broader evidence linking ER crowding to longer waiting times, reduced throughput, and potential deterioration in the quality of care. In addition, more than two-thirds of physicians reported that inappropriate ER use contributes to physician burnout, highlighting the potential workforce implications of sustained crowding and high patient volumes in emergency settings.

In multivariable analysis, emergency physicians were more than twice as likely as family physicians to report that at least half of ER visits are inappropriate. Perceived weaknesses in the referral system and limited access to primary care were also independently associated with this perception. These findings underscore the importance of system-level coordination between primary care and hospital-based services. Strengthening referral pathways, improving access to primary care appointments, and expanding after-hours services may reduce reliance on ERs for nonurgent conditions. In addition, public education initiatives aimed at improving understanding of appropriate care pathways may help guide patients toward the most suitable points of care.

Strengths and limitations

This study has several limitations. First, the use of a convenience sampling approach and voluntary participation may limit the generalizability of the findings and introduce selection bias. Physicians with stronger opinions regarding ER utilization may have been more likely to participate. Second, the study assessed physicians’ perceptions rather than objectively measured patterns of ER utilization, and perceptions may not fully reflect actual clinical appropriateness. Third, although the sample included physicians from multiple practice settings and levels of experience, the cross-sectional design does not permit causal inference regarding factors influencing perceptions of ER use.

Despite these limitations, the study provides important insights into physicians’ views regarding ER utilization within the national health system. By capturing perspectives from both emergency and family medicine physicians, the findings highlight areas of convergence as well as differences in how the two specialties interpret the drivers and consequences of inappropriate ER use. These perspectives may be particularly valuable in the context of ongoing health system reforms aimed at strengthening primary care and improving care coordination.

## Conclusions

In this survey of physicians in Saudi Arabia, inappropriate ER utilization was widely perceived as common and consequential for healthcare delivery. Most respondents reported that a substantial proportion of ER visits are nonurgent and could be managed in primary care settings, with emergency physicians more likely than family physicians to characterize ER use as frequently inappropriate and to report its negative impact on patient safety, delays in care, and physician burnout. Perceived limitations in primary care access and weaknesses in referral systems were strongly associated with these views, highlighting the importance of system-level factors in shaping ER utilization patterns. Strengthening primary care capacity, improving referral pathways, and enhancing public awareness of appropriate care settings may help reduce nonurgent ER visits and support more efficient use of emergency services.

## Appendices

**Table 6:** Survey questionnaire used in the study * Multiple responses were permitted.

Domain	Survey Item	Response Options
Participant characteristics	What is your age group?	<30 years; 30–39 years; 40–49 years; ≥50 years
	What is your sex?	Male; Female
	What is your nationality?	Saudi; Non-Saudi
	What is your specialty?	Family medicine; Emergency medicine
	What is your current professional level?	Resident; Specialist; Consultant
	How many years have you been in clinical practice?	Numeric entry (years)
	Which sector do you primarily practice in?	Government; Private; Other
Perceptions of inappropriate ER utilization	Inappropriate emergency room (ER) use is a major national problem.	Strongly disagree; Disagree; Neutral; Agree; Strongly agree
	A large proportion of ER visits are non-urgent.	Strongly disagree; Disagree; Neutral; Agree; Strongly agree
	Many ER cases could be managed in primary care settings.	Strongly disagree; Disagree; Neutral; Agree; Strongly agree
	Limited access to primary care contributes to ER overuse.	Strongly disagree; Disagree; Neutral; Agree; Strongly agree
	Patients prefer ER care because it is faster and more comprehensive.	Strongly disagree; Disagree; Neutral; Agree; Strongly agree
Perceived causes of inappropriate ER utilization*	In your opinion, what factors contribute to inappropriate ER use?	Limited access to primary care; After-hours service gaps; Poor health literacy; Patient anxiety; Free ER services; Weak referral system
Perceived impact of inappropriate ER use	Inappropriate ER utilization contributes to physician burnout.	Strongly disagree; Disagree; Neutral; Agree; Strongly agree
	Inappropriate ER utilization delays care for critically ill patients.	Strongly disagree; Disagree; Neutral; Agree; Strongly agree
	Inappropriate ER utilization negatively affects patient safety.	Strongly disagree; Disagree; Neutral; Agree; Strongly agree
	Inappropriate ER utilization increases healthcare costs.	Strongly disagree; Disagree; Neutral; Agree; Strongly agree
Overall assessment	In your opinion, what proportion of ER visits are inappropriate?	<25%; 25–49%; ≥50%
